# Supplementary material for: Visual segmentation of complex naturalistic structures in an infant eye-tracking search task
Source: PLoS One. 2022 Apr 1;17(4):e0266158. doi: 10.1371/journal.pone.0266158 (PMC8975119; doi:10.1371/journal.pone.0266158)
Supplement: S1 Text — A. The effect of movement on detection performance. B. Did changes between the differently colored stimuli affect infants’ detection performance? S1 Table: Number of trials per factor level, in the original experiment and in the infant and adult data. (PDF) [file pone.0266158.s001.pdf]

S1 Text. Supplementary results

A. The effect of movement on detection performance

Movement of unconstrained participants during remote-mode eye-tracking substantially affects data quality (e.g., Hessels et al., 2015; Niehorster et al., 2017; Schlegelmilch & Wertz, 2019). However, infants' movements might reflect reactions to the complexity or difficulty of a visual stimulus. We therefore assessed the variable *movement* on the basis of values provided by the eye-tracker, calculated as the maximum of absolute change in head-camera distance within fixations during the presentation of the search stimulus. Movement alone predicted success with $\chi^2(1) = 112$, $p < .001$, in that more movement related to a lower probability to detect the target, logit = -2.41, 95% CI = [-2.86, -1.97]. In the LMM of movement on latency however we diagnosed a skewed error structure, which we corrected by reducing values of movement during hit-trials larger than the 99% percentile ($N = 4$) to the value of the 99% percentile. After correction, movement contributed to the LMM on latency ($\chi^2(1) = 97$, $p < .001$) with stronger movement predicting a longer time to detect a target, $\beta = 1.16$, 95% *CI* = [0.93, 1.39]. We included movement as a covariate in all models, and outlier correction of movement was applied to all models conducted on latency.

B. Did changes between the differently colored stimuli affect infants' detection performance?

In the experiment, the stimuli were alternating in three monochromatic colors [green, red, blue]. However, the perception of brightness varies as a function of hue—two colors with the same luminance level do not necessarily appear equally bright (Helmholtz–Kohlrausch effect). When transforming the stimulus color, we therefore took care that perceived brightness did not vary strongly between the colors by (a) reducing saturation in the HSL

coordinates, thereby decreasing the Helmholtz–Kohlrausch effect, and (b) by using hues of identical distance to each other which were distributed between the pure CMYG or RGB colors (red, yellow, green, cyan, blue, magenta). The pure colors differ more strongly from each other in brightness than the in-between hues. Importantly, during the color transformation, we did not further adapt luminance levels because this might have reduced data quality due to changes in pupil size (Holmqvist et al., 2011).

We tested if stimulus color generally affected search performance. The factor color [red, green, blue] did not predict infants' search performance (success: $\chi^2(2) = 1.6$, *n.s.*; latency: $\chi^2(2) = 3.7$, *n.s.*), nor performance of adults (all $\chi^2(2) < 1.4$, *n.s.*), confirming that the colors we chose to enhance infants' interest in the study did not lead to differences in the detectability of the target.

To further ensure that alternating monochromatic colors between trials did not increase noise in the data, we assessed the effect of these alternations on performance. This showed that infants' performance in trials preceded by an alternating color did not differ from trials with the same color as the previous trial. This was true for the success-hit rate ($M_{change} = .37$, $M_{same} = .36$, t = .2, $p = .85$), and for latency ($M_{change} = 1519$ ms, $M_{same} = 1616$ ms, $t = -.8$, $p = .44$).

S1 Table. Number of trials per factor level, in the original experiment and in the participant data.

Factor	Level	Experiment [a]	Infants	(Hits)	Adults	(Hits)
Background category	Artifact	96	390	(121)	477	(1464)
	N_Element	96	406	(151)	475	(462)
	Vegetation	96	421	(187)	477	(476)
Target category	Artifact	96	401	(151)	477	(472)
	N_Element	96	398	(158)	475	(468)
	Vegetation	96	418	(150)	477	(462)
Category congruency	Congruent	96	402	(147)	475	(468)
	Incongruent	192	815	(312)	954	(934)
Depth congruency	Congruent	144	594	(200)	715	(693)
	Incongruent	144	623	(259)	714	(709)
Location number	1	31	135	(65)	155	(154)
	2	27	120	(54)	134	(132)
	3	31	126	(48)	155	(148)
	4	29	117	(50)	143	(142)
	5	28	113	(16)	138	(134)
	6	24	104	(19)	120	(115)
	7	31	130	(41)	154	(154)
	8	32	143	(65)	159	(159)
	9	27	116	(47)	133	(130)
	10	28	113	(54)	138	(134)
Color	Red	96	410	(141)	477	(471)
	Green	96	398	(155)	475	(466)
	Blue	96	409	(163)	477	(465)

[a] Total of trials in the eight versions of the experiment which were analyzed.
